# Core competencies for scientific editors of biomedical journals: consensus statement

**DOI:** 10.1186/s12916-017-0927-0

**Published:** 2017-09-11

**Authors:** David Moher, James Galipeau, Sabina Alam, Virginia Barbour, Kidist Bartolomeos, Patricia Baskin, Sally Bell-Syer, Kelly D. Cobey, Leighton Chan, Jocalyn Clark, Jonathan Deeks, Annette Flanagin, Paul Garner, Anne-Marie Glenny, Trish Groves, Kurinchi Gurusamy, Farrokh Habibzadeh, Stefanie Jewell-Thomas, Diane Kelsall, José Florencio Lapeña, Harriet MacLehose, Ana Marusic, Joanne E. McKenzie, Jay Shah, Larissa Shamseer, Sharon Straus, Peter Tugwell, Elizabeth Wager, Margaret Winker, Getu Zhaori

**Affiliations:** 1 Centre for Journalology, Clinical Epidemiology Program, Ottawa Hospital Research Institute, 501 Smyth Rd, Room L1248, Box 201B, Ottawa, ON K1H 8L6 Canada; 2 School of Epidemiology, Public Health and Preventive Medicine, Faculty of Medicine, University of Ottawa, Ottawa, Canada; 3 Centre for Journalology, Clinical Epidemiology Program, Ottawa Hospital Research Institute, Centre for Practice-Changing Research (CPCR), Ottawa Hospital - General Campus, Ottawa, Canada; 4 F1000 Platforms, Middlesex House, 34-42 Cleveland Street, London, W1T 4LB UK; 5 Office of Research Ethics and Integrity, Division of Research and Commercialisation and Library, Division of Technology, Information and Library Services, QUT, Brisbane, Australia; 6 Department of Strategy, Policy and Information, World Health Organization, Geneva, Switzerland; 7 American Academy of Neurology, St. Paul, Minnesota USA; 8 Council of Science Editors, Denver, Colorado USA; 9 Department of Health Sciences, University of York, York, UK; 10 Cochrane Central Executive, London, UK; 11 Department of Psychology, University of Stirling, Stirling, UK; 12 American Congress of Rehabilitation Medicine, Reston, Virginia USA; 13 The Lancet, London, UK; 14 Institute of Applied Health Research, College of Medical and Dental Sciences, University of Birmingham, Birmingham, UK; 15 JAMA and The JAMA Network, Chicago, Illinois USA; 16 Department of Clinical Sciences, Liverpool School of Tropical Medicine, Liverpool, UK; 17 Division of Dentistry, School of Medical Sciences, University of Manchester, Manchester, UK; 18 BMJ Open, London, UK; 19 University College London, London, UK; 20 Shiraz University of Medical Sciences, Shiraz, Iran; 21 R&D Headquarters, Petroleum Industry Health Organization, Shiraz, Iran; 22 World Association of Medical Editors (WAME), Chicago, Illinois USA; 23 Elsevier, Philadelphia, Pennsylvania USA; 24 Canadian Medical Association Journal, Ottawa, Canada; 25 Department of Otorhinolaryngology, College of Medicine — Philippine General Hospital, University of the Philippines Manila, Manila, Philippines; 26 Philippine Association of Medical Journal Editors (PAMJE), Quezon City, Philippines; 27 Asia Pacific Association of Medical Journal Editors (APAME), Manila, Philippines; 28 Cochrane Editorial Unit, London, UK; 29 University of Split School of Medicine, Cochrane Croatia Editor, Journal of Global Health, Split, Croatia; 30 European Association of Science Editors, http://www.ease.org.uk/; 31 School of Public Health and Preventive Medicine, Monash University, Melbourne, Australia; 32 School of Medicine, Patan Hospital, Kathmandu, Nepal; 33 Patan Academy of Health Sciences, Kathmandu, Nepal; 34 Nepal Association of Medical Editors, Kathmandu, Nepal; 35 Department of Medicine, University of Toronto, Toronto, Canada; 36 Department of Medicine, Faculty of Medicine, University of Ottawa, Ottawa, Canada; 37 Clinical Epidemiology Program, Ottawa Hospital Research Institute, Ottawa, Canada; 38 Sideview, Princes Risborough, UK; 39 TRIBE Doctoral School, University of Split School of Medicine, Split, Croatia; 40 Chinese Medical Journal, Beijing, China

**Keywords:** Core competencies, Scientific editor, Biomedical journal, Delphi, Expert consensus, Editor role

## Abstract

**Background:**

Scientific editors are responsible for deciding which articles to publish in their journals. However, we have not found documentation of their required knowledge, skills, and characteristics, or the existence of any formal core competencies for this role.

**Methods:**

We describe the development of a minimum set of core competencies for scientific editors of biomedical journals.

**Results:**

The 14 key core competencies are divided into three major areas, and each competency has a list of associated elements or descriptions of more specific knowledge, skills, and characteristics that contribute to its fulfillment.

**Conclusions:**

We believe that these core competencies are a baseline of the knowledge, skills, and characteristics needed to perform competently the duties of a scientific editor at a biomedical journal.

## Introduction

Scientific editors (editors are responsible for the content and policies of journals, and scientific editors are members of the team who contribute to that process by virtue of their scientific knowledge and experience) are responsible for deciding which articles to publish in biomedical journals [1]. A scoping review of the skills and requirements for scientific editors at biomedical journals carried out by some of the authors of this paper showed that most of the literature that contained recommendations on this issue was not research-based [2]. Rather, recommendations were documented in position papers and in guidance for members of editor organizations [3–8]. While many of these publications have offered perspectives on the knowledge, skills, and characteristics needed to be an effective scientific editor, there appears to be no consensus on which of these are fundamental to the scientific editor role. To our knowledge, no formal set of core competencies for this group has been established locally or globally. Our aim was to develop a minimum set of core competencies for scientific editors of biomedical journals.

## Developing the core competencies

We used an integrated knowledge translation approach [9, 10] to engage stakeholders in a consensus-based process to develop a minimum set of core competencies for scientific editors of biomedical journals that was informed by a scoping review and editors’ perspectives. At the program outset, the team from the Centre for Journalology at the Ottawa Hospital Research Institute (JG, DM, KDC, and LS) assembled a core group of experts to represent scientific editing and publisher stakeholder groups. The experts included scientific editors from different parts of the world and various types and sizes of journals, editors-in-chief, and representatives from editorial organizations, biomedical journals, and publishers (Table 1). Our goal was to include diverse perspectives representing the spectrum of work involved in scientific editing.

**Table 1:** List of participating stakeholder groups

Asia Pacific Association of Medical Journal Editors (APAME)	
BioMed Central (BMC)	
*British Medical Journal (The BMJ)*	
*Canadian Medical Association Journal (CMAJ)*	
*China Medical Tribune (CMT)*	
Cochrane	
Committee on Publication Ethics (COPE)	
Council of Science Editors (CSE)	
Elsevier	
Eastern Mediterranean Association of Medical Editors (EMAME)	
European Association of Science Editors (EASE)	
Nepal Association of Medical Editors (NAME)	
Philippine Association of Medical Journal Editors (PAMJE)	
World Association of Medical Editors (WAME)	
World Health Organization (WHO)	

We followed a three-step process to develop the core competencies, which is followed by a fourth step to be implemented post-publication:

1. Pre-meeting activities (conduct scoping review and environmental scan; survey of editors’ perceptions/training needs; modified Delphi exercise)
2. Face-to-face consensus meeting (present results of pre-meeting research; hold consensus-based discussions)
3. Post-meeting activities (finalize competencies; solicit feedback from managing editors; survey editors for usefulness of competencies)
4. Post-publication activities (seek endorsement; plan for dissemination and implementation activities)

### Pre-meeting activities

#### Scoping review and environmental scan

**Fig. 1:**
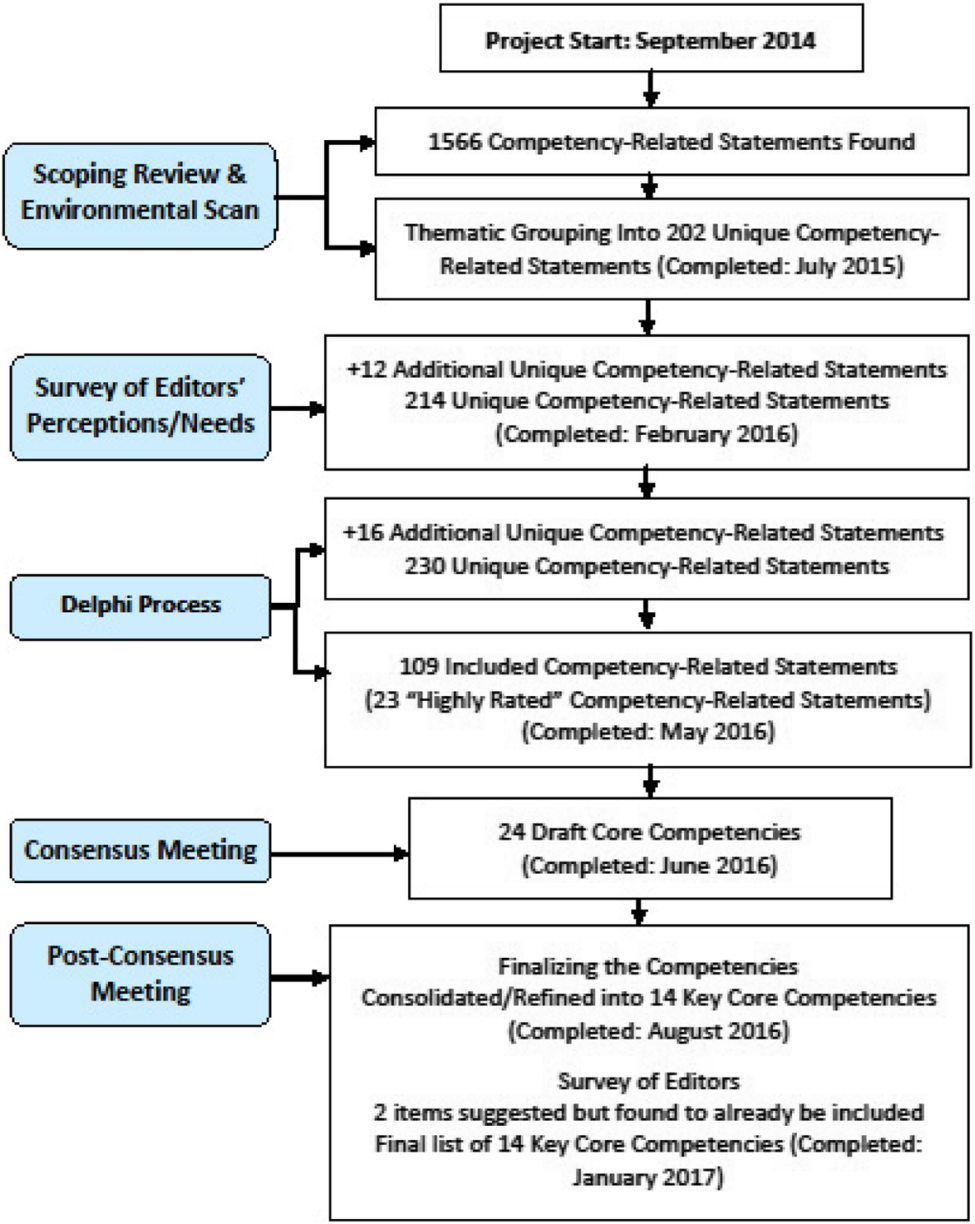
Flow diagram for core competency development

A subset of authors from the current publication (VB, PB, SB-S, KDC, JD, JG, PG, HM, DM, LS, SS, PT, EW, and MW) conducted a scoping review and environmental scan of the literature related to core competencies for scientific editors [2]. This included a review of the published and unpublished scientific and non-scientific literature that contained competency-related statements pertaining to scientific editors. They found a total of 225 full-text documents, 25 of which were research articles. From the 225 documents, they extracted a total of 1566 statements possibly related to core competencies for scientific editors of biomedical journals, which ultimately produced a list of 202 unique competency-related statements after de-duplication [2] (Fig. 1).

#### Survey of editors’ perceptions and training needs

Another subset of authors from the current publication (VB, PB, SB-S, KDC, JD, JG, PG, DM, LS, SS, PT, and MW) engaged stakeholder organizations by inviting their scientific editor members to participate in an online survey of editors’ perceptions and their training needs [11]. The participants were respondents to advertisements seeking current or former scientific editors of journals. Advertisements for the research were sent to organizations having a large scientific editor membership (e.g., World Association of Medical Editors [WAME], Council of Science Editors [CSE], European Association of Science Editors [EASE], Cochrane), who forwarded an announcement about the survey to their membership. They collected demographic data and invited respondents to share their perceptions of the relevance of competency-related statements in their role as editors. They also asked respondents to share their perceptions of their own competence related to these statements. There were 38 statements, developed based on data collected in our scoping review [2] and from input from the publication’s authors. These statements were chosen to broadly cover major areas associated with the scientific editor role, including editors’ knowledge, expertise, skills, and experience. Finally, they asked respondents to create a ranked list of their training needs. A total of 148 participants from around the world contributed to the needs assessment survey. The ranked list of needs provided an additional 12 unique competency-related statements that were not previously included in the scoping review and environmental scan (Fig. 1). This provided valuable insight into the views and needs of scientific editors from different demographics and circumstances in the journal publishing landscape.

#### Modified Delphi process

A final subset of authors from the current publication (VB, PB, SB-S, KDC, JD, JG, PG, DM, LS, SS, PT, and MW) invited the respondents from the editor survey to participate in a three-round modified Delphi process to rate the importance of the 214 competency-related statements arising from the scoping review, environmental scan, and editor survey [2]. During the first round of the Delphi, they also invited participants to suggest any missing items, from which a further 16 unique items were found, bringing the total number of competency-related statements to 230. A total of 105 participants participated in the Delphi, with 27 of them completing one round, 20 completing two rounds, and 58 participants completing all three rounds. Their responses produced a list of 23 “highly rated” and 86 other “included” competency-related statements to help inform the decision-making process during the consensus meeting (Fig. 1). (The manuscript describing this process and the survey of editors’ perceptions and training needs [11].)

### Face-to-face consensus meeting

In early June 2016, the Centre for Journalology group, in consultation with the other authors of the pre-meeting activities publications, assembled a group of 23 stakeholders in Strasbourg, France for a one-and-a-half-day meeting to work towards a minimum set of core competencies for scientific editors of biomedical journals. This group included nine stakeholders previously involved in the program (PB, SB-S, JG, PG, HM, DM, PT, EW, and MW) and 13 new stakeholders (SA, KB, JC, AG, KG, FH, SJ, DK, JL, AM, JM, JS, and GZ). The group was purposively sampled using snowballing principles; we invited our core group of experts to attend the consensus meeting and also asked them to contribute the names of other relevant editors (and others) who could potentially represent a range of perspectives, for example, due to their geographical location, size and type of journal where they work, experience with the publishing process, etc.). Participants were invited via a formal letter of invitation emailed by the lead author. We did not specifically solicit representatives of author and peer reviewer groups, as most of the consensus meeting participants were, or had been at one time, authors and/or peer reviewers and therefore could provide insight concerning these perspectives. The results of the scoping review and environmental scan, survey of editors’ perceptions and training needs, and modified Delphi were presented to the group. The presentation was followed by focused discussions on the 23 highly rated competency-related statements resulting from the Delphi, which were divided into four broad categories. Within these discussions, the group identified the competency-related statements that represented core competencies and suggested how to improve each statement. Other competency-related statements from the list of 86 included statements were also considered. Following these discussions, the selected core competencies were reviewed to determine whether there were any missing competencies. At the conclusion of the consensus meeting, the group emerged with a draft list of 24 core competencies (Fig. 1).

### Post-consensus meeting activities

#### Finalizing the competencies

Following the consensus meeting, numerous email rounds of editing and feedback took place among consensus meeting participants (led by JG), stakeholders who did not attend the consensus meeting (KDC, JD, LS, and SS), and other stakeholders who were invited to the consensus meeting but were unable to attend (VB, LC, and TG). After removing redundancies and overlap between items, combining similar items, refining wording, and removing items after further discussion, the group finally arrived at a final set of 14 core competencies for scientific editors of biomedical journals (Table 2).

**Table 2:** Minimum set of core competencies for scientific editors of biomedical journals

A. Editor qualities and skills	
Key competencies	Elements
Scientific editors are able to:	
1. Demonstrate experience and broad knowledge of the field(s) covered by the journal	1.1 Identify situations in which the knowledge or skill required exceeds their level of competency and seek help or advice from appropriate colleagues or organizations 1.2 Possess a knowledge base that includes training and/or experience in a research environment (applies only to editors working with research-based manuscripts)
2. Synthesize information and views from a wide range of sources and make informed decisions	2.1 Exercise sound judgment in making editorial decisions 2.2 Make fast, considered decisions about manuscripts and any other issues that require a response 2.3 Reconsider decisions when necessary and respond promptly and appropriately to complaints
3. Practice lifelong learning related to their role as an editor and within their area(s) of expertise	3.1 Set personal learning goals and work to fulfill them 3.2 Maintain current knowledge related to important developments and trends in their respective area(s) of expertise 3.3 Join a professional society for editors and/or participate in continuing education offerings for editors
4. Communicate clearly and effectively manage communications and relationships with authors, peer reviewers, other editors, staff (if applicable), readers, journal owners, publishers, and other relevant individuals or groups	4.1 Provide clear editorial instructions to authors and peer reviewers 4.2 Ensure appropriate and effective use of communication, including correspondence, email, and social media 4.3 Describe the roles and responsibilities of editorial staff (if applicable) 4.4 Mentor, educate, train, and provide feedback to other editors and staff when needed (if applicable) 4.5 Identify and apply the journal’s policies regarding embargos and relations with news media
5. Act with leadership and integrity and be accountable to authors, peer reviewers, fellow editors, readers, journal owners, publishers, and other relevant individuals and groups	5.1 Demonstrate skill, tact, diplomacy, confidentiality, and professionalism in interactions with authors, peer reviewers, readers, staff (if applicable), and other relevant individuals or groups, particularly when concerns or disputes arise regarding the peer review and publication process 5.2 Monitor and safeguard the fairness, timeliness, thoroughness, confidentiality (as appropriate), and courtesy in the processing of manuscripts and in responding to queries from authors and reviewers
B. Publication ethics and research integrity	
Key competency	Elements
Scientific editors are able to:	
1. Demonstrate knowledge related to the integrity of research and publishing and apply best practices in dealing with research or publication misconduct, misbehavior, and questionable practices	1.1 Describe what constitutes a breach in publication ethics, act on allegations of misconduct, misbehavior, or questionable practices, and proceed to issue an erratum or retraction when it is warranted, maintaining confidentiality, fairness, and due process 1.2 Identify and assess problems related to selective reporting of publications, outcomes, and analyses 1.3 Identify conflicts of interest for authors, editors, peer reviewers, publishers, and funders (of journals, authors, or research) in relation to scientific reports, opinion pieces, reviews, and other article types, and implement transparent policies to disclose these effectively 1.4 Identify and appropriately manage redundant (or duplicate or repetitive) submissions and publications 1.5 Identify and appropriately address bias in the reporting, interpretation, and extrapolation of study findings 1.6 Identify and enforce policies related to reproducible research, data availability, and registration of clinical trials, systematic reviews, and protocols 1.7 Identify and ensure that appropriate reporting guidelines have been adhered to by authors and peer reviewers 1.8 Articulate the importance of dialogue and contestation following the publication of research and help ensure the opportunity for and moderation of these debates (including post-publication criticisms of research, seeking authors’ responses, corrections, or retractions, and publishing as appropriate, to correct the scientific record) 1.9 Identify and apply the principles of confidentiality and anonymity in the peer review and editorial processes (as they apply to their journal)
2. Identify and uphold the principles of ethical research involving humans and animals when appraising manuscripts	2.1. Ensure that the laws and ethical standards are followed regarding respect, privacy, informed consent for participation in research, protection of individual participant data described in publications, and reporting of review and/or waiver of review by ethics committees or institutional review boards of all studies involving human participants or animals 2.2. Identify issues related to ”dual-use research of concern” (i.e., research that could be directly misapplied to pose a substantial threat to public health, safety, or security, agricultural crops and other plants, animals, the environment, or materials)
3. Articulate and apply their responsibilities and rights as a journal editor	3.1. Identify and comply with copyright and licensing regulations 3.2. Identify and comply with libel law, as it pertains to the jurisdiction where the journal is published 3.3. Identify and adhere to the principles of editorial independence in relation to journal owners and journal publishers while recognizing their legal responsibilities in regard to them 3.4. Identify and adhere to the principles of editorial integrity, including policies and procedures to ensure fairness to authors, peer reviewers, and readers 3.5. Help ensure that journal advertising policy adheres to best practices 3.6. Disqualify themselves from the editorial decision-making process when potential or actual conflicts of interest pertaining to them arise
C. Editorial principles and processes	
Key competencies	Elements
Scientific editors are able to:	
1. Identify and use trustworthy resources	1.1 Identify and use resources that describe best practices related to scholarly publishing, publication ethics, and technical editing for authors, editors, and peer reviewers
2. Select journal content that reflects the goals and scope of the journal	2.1 Identify the vision and mission (aim and scope) of their journal and determine whether submitted manuscripts align with them
3. Analyze journal policies, practices, and performance metrics to improve journal performance	3.1 Interpret journal and scholarly metrics and ensure that these metrics are not manipulated in a way that is unfair or unscrupulous 3.2 Use feedback from readers and metrics to help ensure the journal meets readers’ needs 3.3 Analyze journal performance metrics such as time from submission to first decision, time to acceptance, and time to publication, and identify specific steps to reduce unnecessary delays 3.4 Explain journal workflows and publication models
4. Evaluate the scientific rigor and integrity of manuscripts and make editorial decisions after consideration of reviewers’ and other editors’ comments	4.1 Check the content of manuscripts submitted for publication for completeness, logic, and consistency 4.2 Assess the appropriateness of the research design and methods described in research manuscripts, as well as the validity of findings and conclusions, in relation to the stated research question 4.3 Form rational preliminary opinions on the relevance of a submitted manuscript to the journal based on the journal’s aims and scope and the quality of the submission 4.4 Articulate to authors and enforce the journal’s policy on attributing authorship and contributorship, conflict of interest disclosures, disclosure of funding sources, and requirements for quality of reporting 4.5 Ensure clarity, balance, and use of appropriate sources for arguments and recommendations made in manuscripts 4.6 Provide timely feedback that synthesizes views of reviewers and editors and identifies critical points to help authors make improvements 4.7 Triage manuscripts thoughtfully and in a timely manner (for journals that use such a process)
5. Apply best practices for research and other manuscript presentation when evaluating and requesting revision of manuscripts	5.1 Recognize and apply best practices in evaluating different types of manuscripts, including research-based and non-research (e.g., opinion pieces, clinical education articles) manuscripts 5.2 Identify and apply best practices in evaluating adherence to the principles of research question/hypothesis development and different types and levels of evidence 5.3 Identify and apply best practices in evaluating adherence to the principles of clinical research design (if applicable) and quantitative and/or qualitative research methods (as appropriate) 5.4 Identify and apply best practices in assessing the appropriateness of and evaluating the use of basic statistics (if applicable) 5.5 Identify and apply best practices in evaluating the presentation of research data and parts, purposes, and characteristics of tables, charts, graphs, images, multimedia, and data supplements 5.6 Identify and apply best practices in evaluating citations and references
6. Manage and assure the integrity of the peer review process	6.1 Describe different models of peer review 6.2 Select peer reviewers who possess the appropriate expertise needed to review a manuscript thoroughly 6.3 Identify and exclude (as appropriate) peer reviewers with potential conflicts of interest 6.4 Justify recommended manuscript changes based on peer reviewers’ comments and journal policy 6.5 Provide tactful feedback to peer reviewers on their performance 6.6 Assess the quality of, and maintain performance statistics on, peer reviewers to avoid re-inviting excessively tardy and/or poor reviewers 6.7 Regularly express gratitude toward peer reviewers for their service and offer incentives and rewards as appropriate (e.g., continuing education credit, complimentary or discounted access to the journal) 6.8 Ensure that the peer review of a manuscript proceeds with minimal additional delay when reviewers fail to submit a timely review 6.9 Regularly monitor and audit the journal’s performance in terms of acceptance and rejection rates, percentage of papers undergoing peer review, the percentage of peer reviewers agreeing to review, and turnaround

#### External validation

We also asked two managing editors (one not involved in this initiative) to review the proposed competencies, and we incorporated their feedback into the refining process. The managing editor of *The Journal of the American Medical Association (JAMA)* and Jason Roberts of *Headache: The Journal of Head and Face Pain* are responsible for facilitating the peer review operations of their respective journals, the implementation of editorial policies and procedures, and ensuring that accepted manuscripts are formatted to fit the needs of the publisher.

#### Survey of editors on the usefulness of the core competencies

After reaching agreement on the final version of the competencies, we solicited the feedback of scientific editors from a small (*Headache*) and a medium-sized (*Canadian Medical Association Journal [CMAJ]*) journal. These editors were asked to take 2–3 weeks to consider and reflect on the relevance of the competencies in the context of their role as a scientific editor. Eight editors answered a short survey (hosted on SurveyMonkey.com) asking about the usefulness, aspirational qualities, and relevance of the competencies and whether any important competencies were missing. Their answers were generally supportive of the competencies as useful and relevant and somewhat mixed on their aspirational qualities. Two new items were suggested, which were later determined to already be included in the list of core competencies.

## The core competencies for scientific editors of biomedical journals

Table 2 displays the final minimum set of core competencies for scientific editors of biomedical journals. It contains 14 key core competencies divided into three major areas. Each competency has a list of associated elements or descriptions of more specific knowledge, skills, and characteristics that contribute to the fulfillment of the associated core competency. These elements are meant to be illustrative examples of the key competencies rather than a comprehensive breakdown of the competencies.

## Scope of the core competencies

We have made extensive efforts to produce consensus-based, end-user informed core competencies for scientific editors of biomedical journals that are driven by a scoping review and informed by end users. However, we acknowledge the limits of their scope as well. Specifically, we attempted to identify only the competencies that would be applicable across the entire spectrum of scientific editors of biomedical journals, regardless of journal size, type, geographic location, publishing model, or any other defining characteristic.

In some instances, there were important elements that we believed should be included, with the recognition that they may not apply to all scientific editors of biomedical journals in all situations. Therefore, we have inserted conditional language (e.g., “if applicable”, “as appropriate”) into some of the competency statements. It is possible that some other statements without the conditional language may also not be applicable or advisable given a scientific editor’s specific circumstances. In these cases, editors should act in the spirit of the competency instead of the literal description. Conversely, we also expect that each individual scientific editor position will potentially involve more competencies and/or elements than are identified in this list to address the particularities of the role and the characteristics of the journal. We encourage editors to identify these additional competencies and elements in order to complete the core competency profile for their particular role.

Since these core competencies are directed to editors of biomedical journals, they may not apply as well to editors in other scientific disciplines or domains outside of the scientific realm. It would be important to test these competencies with scientific editors in other fields to better understand their applicability in other disciplines. Although these core competencies can and should be applicable to those who hold the role of editor-in-chief, it is important to note that any competencies related exclusively to the editor-in-chief position were purposely removed from this list, as they do not necessarily apply to all scientific editors. We encourage other editorial groups and members of the editor-in-chief community to collaboratively create extensions to this list that address their more specialized role.

We took considerable care in crafting the specific language used to describe each competency, including trying to preserve the original language used in the scoping review, environmental scan, needs assessment, and modified Delphi, whenever possible. However, some of this language may be open to varying interpretations; thus, we hope to clarify any language issues in an upcoming explanation and elaboration document on each of the key competencies and their associated elements.

### Post-publication activities

#### Endorsement

With the core competencies now established, we have begun the process of seeking a formal statement of endorsement from our stakeholders which will be used when promoting the competencies. At the time of submission, the competencies have been formally endorsed by Cochrane, EASE, and the Asia Pacific Association of Medical Journal Editors (APAME). The remaining editorial organizations on our stakeholder list (Table 1) are in the process of considering the core competencies for endorsement.

#### Dissemination

A subset of our authors will form a small committee that is tasked with developing a strategy to effectively disseminate the core competencies worldwide. At the time of publication, the core competencies have already been presented at the 2016 APAME conference and the 2017 CSE annual meeting and during an invited talk at the 2017 International Congress on Peer Review and Scientific Publication.

#### Implementation

Another subset of our authors will form a small committee to address how to best implement the core competencies. Editors, their publishers, and editorial groups who endorse these core competencies may be wondering how best to implement them. We believe it will be important to tailor training against each core competency described above. Some high-quality training might already exist for some competencies, while training for others will likely require development. Most editors are geographically dispersed, and it might be most effective to consider online training to maximize reach.

#### Evaluation

The process of developing the core competencies is similar to that of developing any intervention. As with any intervention, it will be important to address whether implementation of these core competencies is associated with improvements in the roles and functions of scientific editors, such as increased mentorship within a journal and applying best practices in evaluating adherence to research methods of submitted manuscripts [12]. One strong evaluation option is to consider an experimental design whereby some journals expose their scientific editors to formal core competency training while other journals act as a ”standard practice” control. The details of any evaluation require further deliberation and engagement. The recently established Best Practice Journal Research Network is one possible group to conduct such a study [13].

## Discussion

The need for consistent, core competencies in scientific editing is clear. Proponents of the reducing waste in research campaign, for example, say that the system of assessing quality of scientific research needs improvement [14]. Scientific editors are clearly central to that system, which the Declaration of Helsinki recognizes by noting the responsibilities of editors in ensuring the highest possible standards in what is published in their journals [15]. Specifically developed for scientific editors of biomedical journals, these core competencies establish a baseline for the knowledge, skills, and characteristics needed in order to competently perform the duties of a scientific editor. In essence, they describe the agreed-upon minimum criteria for effectively performing the duties of a scientific editor at a biomedical journal. Our focus was on developing an intervention (i.e., core competencies) to help scientific editors. While scientific editors are central to helping improve the publication record, there are other constraints in the system on which they have limited influence. Most researchers find themselves in a ”publish or perish” environment. Academic institutions typically assess their faculty for promotion and tenure based on bibliometrics (i.e., some form of counting publications), which are often misaligned with societal needs. Quantity may be given undue priority over quality. Some researchers may “short-circuit” the quality of their research to meet publication needs, which can be difficult or impossible for editors to detect [16]. The push for quantity has also resulted in some authors circumventing peer review and editorial oversight to achieve publication [17].

The competencies themselves are not novel or new, per se, to the published literature. They were derived from our previous scoping review of existing published (and unpublished) competency statements, and despite having the opportunity throughout the process to add other competencies that were not derived from our comprehensive evidence-gathering process (e.g., scoping review, Delphi exercise), no completely novel competencies emerged. Likewise, the core competencies, in general, do not appear to be novel to most of the editors we surveyed. In comparing the findings from our survey of scientific editors of biomedical journals' training needs, perceptions of competence, and ratings of importance of competency-related statements, we found a high degree of congruency between the core competencies presented in this manuscript and editors’ needs and ratings of importance. In fact, both the top five editor training needs and the six competency-related statements rated most important are all found within the 14 core competencies and/or their elements. These areas include: statistics, research methods, publication ethics, the peer review process, integrity/professionalism, good decision-making, language skills, and journal indexing. However, presented here the competencies represent a new level of collaboration and rigor in their development for scientific editors of biomedical journals. To our knowledge, these core competencies did not exist in the peer-reviewed literature previously.

Although we used a rigorous, consensus-based approach in developing these core competencies, our methods nevertheless have limitations. Time and resource constraints, limited participation, and differences in participants’ perceptions, experiences, and interpretations may also have influenced the process of developing these competencies. In addition, the restriction of the consensus meeting and post-consensus meeting participation mainly to individuals representing editors and publishers may have limited inclusion of perspectives of other relevant groups (e.g., authors, readers, peer reviewers) in the selection and wording of the core competencies. However, the editors involved in the process were also authors and peer reviewers previously or currently. All participants are also readers. As such, these perspectives were not completely lost.

These core competencies might also be useful to other types of editors at biomedical journals, such as technical editors (i.e., those responsible for substantial editing of manuscripts, including re-writing for clarity and language), and to editors in other disciplines. Some editors and publishers might find these competencies simply aspirational, while others may want to recommend their implementation. We encourage stakeholders in the biomedical (and other) domain(s) to collaborate with each other to develop extensions (or modifications) to these core competencies to address the specific needs of particular groups of editors (such as scientific editors at small or large journals, editors-in-chief, or scientific editors in other disciplines), much in the same way that extensions to the Consolidated Standards of Reporting Trials (CONSORT) reporting guideline have been created to address the reporting of specific types of trials [18].

The aim of our program to develop core competencies was to provide guidance to scientific publishers and editors of biomedical journals worldwide on the minimum knowledge, skills, and characteristics that are needed to be effective in their role. We emphasize that this list of core competencies is meant to represent the minimum standards for the role of scientific editor, regardless of the particularities of each journal. We acknowledge there may be other essential competencies that relate to scientific editors, depending on their specific circumstances.

The immediate short-term goal of this program was to develop an essential set of core competencies and examples and to encourage endorsement across a broad spectrum of journals and editorial groups. A subsequent short-term goal is to develop training modules based on these core competencies. Another short-term goal of this program is to develop a core competency-based curriculum with which to train scientific editors of biomedical journals. Once the curriculum is completed, evaluating the competencies will be essential. We hope these short-term goals will help scientific editors improve their journals and the publication record. A longer term goal is to consider a certification process whereby journal editors can obtain official recognition for demonstrating that they possess all of the core competencies. This process would also allow journals and publishers a way to distinguish themselves as having ensured a minimum standard of competency among all of their scientific editors, possibly through a system of digital badges [19]. The downstream consequences of these efforts might include an increase in the research value of science and a higher quality of scientific publications.

## Abbreviations

APAME: Asia Pacific Association of Medical Journal Editors
BMC: BioMed Central
BMJ: British Medical Journal
CMAJ: Canadian Medical Association Journal
CMT: China Medical Tribune
CONSORT: Consolidated Standards of Reporting Trials
COPE: Committee on Publication Ethics
CSE: Council of Science Editors
EASE: European Association of Science Editors
NAME: Nepal Association of Medical Editors
PAMJE: Philippine Association of Medical Journal Editors
WAME: World Association of Medical Editors
WHO: World Health Organization

## References

[1] Kleinert S, Wager E, Mayer T, Steneck N (2011). Responsible research publication: international standards for editors. A position statement developed at the 2nd World Conference on Research Integrity, Singapore, July 22-24, 2010. Promoting research integrity in a global environment.

[2] Galipeau J, Barbour V, Baskin P, Bell-Syer S, Cobey K, Cumpston M, Deeks J, Garner P, MacLehose H, Shamseer L, Straus S, Tugwell P, Wager E, Winker M, Moher D (2016). A scoping review of competencies for scientific editors of biomedical journals. BMC Med.

[3] Council of Science Editors: White paper on publication ethics: CSE’s white paper on promoting integrity in scientific journal publications, 2012 update. http://www.councilscienceeditors.org/resource-library/editorial-policies/white-paper-on-publication-ethics/. Accessed 15 Dec 2016.

[4] International Committee of Medical Journal Editors: Recommendations for the conduct, reporting, editing, and publication of scholarly work in medical journals. Updated December 2016. http://www.icmje.org/recommendations. Accessed 15 Dec 2016.

[5] Committee on Publication Ethics: Code of conduct and best practice guidelines for journal editors, Version 4. http://publicationethics.org/files/Code%20of%20Conduct_2.pdf. Accessed 15 Dec 2016.

[6] Flanagin A. Editorial responsibilities, roles, procedures, and policies. In: AMA manual of style: a guide for authors and editors. 2007. http://www.amamanualofstyle.com/view/10.1093/jama/9780195176339.001.0001/med-9780195176339-div1-68. Accessed 15 Dec 2016.

[7] World Association of Medical Editors: Syllabus for prospective and newly appointed editors. http://www.wame.org/about/syllabus-for-prospective-and-newly-appointed. Accessed 12 Dec 2016.

[8] European Association for Scientific Editing (EASE) (2013). Science editor's handbook.

[9] Kothari A, Wathen CN (2013). A critical second look at integrated knowledge translation. Health Policy..

[10] Bowen S, Graham ID, Straus SE, Tetroe J, Graham ID (2013). Integrated knowledge translation. Knowledge translation in health care: moving from evidence to practice.

[11] Galipeau J, Cobey KD, Barbour V, et al. An international survey and modified Delphi process revealed editors’ perceptions, training needs, and ratings of competency-related statements for the development of core competencies for scientific editors of biomedical journals [version 1; referees: awaiting peer review]. F1000Research 2017;6:1634. 10.12688/f1000research.12400.1 PMC5605946 28979768

[12] Hopewell S, Boutron I, Altman DG, Barbour G, Moher D, Montori V, Schriger D, Cook J, Gerry S, Omar O, Dutton P, Roberts C, Frangou E, Clifton L, Chiocchia V, Rombach I, Wartolowska K, Ravaud P (2016). Impact of a web-based tool (WebCONSORT) to improve the reporting of randomised trials: results of a randomised controlled trial. BMC Med.

[13] Moher D, Ravaud P (2016). Increasing the evidence base in journalology: creating an international best practice journal research network. BMC Med.

[14] Kleinert S, Horton R (2014). How should medical science change?. Lancet.

[15] Declaration of Helsinki. https://www.wma.net/policies-post/wma-declaration-of-helsinki-ethical-principles-for-medical-research-involving-human-subjects/. Accessed 17 Dec 2016.

[16] Fanelli D (2010). Do pressures to publish increase scientists' bias? An empirical support from US states data. PLoS ONE.

[17] Shamseer L, Moher D, Maduekwe O, Turner L, Barbour V, Burch R, Clark J, Galipeau J, Roberts J, Shea BJ (2017). Potential predatory and legitimate biomedical journals: can you tell the difference? A cross-sectional comparison. BMC Med.

[18] EQUATOR Network. Search for reporting guidelines. http://www.equator-network.org/?post_type=eq_guidelines&eq_guidelines_study_design=0&eq_guidelines_clinical_specialty=0&eq_guidelines_report_section=0&s=+CONSORT+extension&btn_submit=Search+Reporting+Guidelines. Accessed 17 Dec 2016.

[19] Kidwell MC, Lazarevi LB, Baranski E, Hardwicke TE (2016). Badges to acknowledge open practices: a simple, low-cost, effective method for increasing transparency. PLoS Biol.

